# Image-based angio-adaptation modelling: a playground to study cerebrovascular development

**DOI:** 10.3389/fphys.2023.1223308

**Published:** 2023-07-26

**Authors:** Rui D. M. Travasso, Vanessa Coelho-Santos

**Affiliations:** ^1^ Department of Physics, Center for Physics of the University of Coimbra (CFisUC), University of Coimbra, Coimbra, Portugal; ^2^ Coimbra Institute for Biomedical Imaging and Translational Research (CIBIT), University of Coimbra, Coimbra, Portugal; ^3^ Institute for Nuclear Sciences Applied to Health (ICNAS), University of Coimbra, Coimbra, Portugal

**Keywords:** vascular biology, mathematics, modelling, neurodevelopment, neurovascular

Brain development is a complex process that involves a cerebrovascular shifting landscape with marked expansion and refinement for metabolic homeostasis. This cerebrovascular system is a 3D network composed of arteries, capillaries, and veins (Coelho-Santos and Shih, 2020). Remarkable in several ways, the capillary network that forms the blood brain barrier (BBB), conferring brain protection from the periphery, it is also responsible for most of the nutrients and wastes exchange. Indeed, the wellbeing of the cerebrovascular network is essential to keep the metabolic homeostasis across the lifespan, by matching the brain’s high energy demand with the supply of energy substrates and oxygen from the blood, through a process called neurovascular coupling (Iadecola, 2017).

Cerebrovascular network architecture is not predetermined, but a result of biological responses (angiogenesis, remodelling, and pruning) of the vascular endothelial cells to the stimuli that they receive. During brain development, the growth of the initial vascular plexus, that resulted from vasculogenesis, occurs by angiogenesis (Crouch et al., 2022; Coelho-Santos et al., 2021), resulting in an increase of the local nutrient and oxygen supply at the demand of the active neural tissue. Neuronal and vascular systems grow in parallel and seem influence each other (Lacoste et al., 2014). This initial angiogenesis expansion of the vascular plexus is followed by a remodelling process driven by the blood flow and shear stress: the increase in shear stress promotes endothelial cell proliferation (Pries et al., 2015) and higher blood pressures promote vascular smooth muscle cell proliferation (Loerakker et al., 2018), leading to vessel widening. On the other hand, in already oxygenated regions or in vessels where the flow is very low, endothelial cells return to the parent’s vessels through pruning and vascular regression (Franco et al., 2015). Along this journey, mural cells (smooth muscle cells and pericytes) (Coelho-Santos et al., 2021; Crouch et al., 2022) and neurons (Lacoste et al., 2014) are already in intimacy contact within the endothelium participating in the expansion of the vascular system. However, other cells like astrocytes are recruited later to join the vasculature providing quiescence and maturation (Gilbert et al., 2019). Altogether, these cells constitute a functional neurogliovascular unit. Therefore, neurovascular coupling, the communication between neurons and the vascular system, becomes efficient after birth (Kozberg et al., 2016).

Understanding cerebrovascular network development has been challenging due to its complexity and poor accessibility during development. Much of the important knowledge we have nowadays about vascular network development has been gathered from studies done in zebrafish, heart or retina (Franco et al., 2015; Pries et al., 2015). Although some characteristics of the vascular systems may be common in different organs due to their similarities, we need to individualise this system to each organ, namely, the mammalian brain. Brain endothelium has particular features such as BBB and consequently its vascular system is unique. Our insight about the mammalian cerebrovascular network development is still evolving. Just recently, we have opened the “window” to explore the cortical cerebrovascular development in the neonatal brain (Harb et al., 2013; Letourneur et al., 2014; Coelho-Santos et al., 2021). The appearance of cutting-edge techniques such as multiphoton microscopy allowed the longitudinal study in greater detail of the capillary network construction (Coelho-Santos et al., 2021). Multiphoton microscopy is a preferred method to image *in vivo* dynamic cellular processes such as neurovascular coupling due its high resolution and low toxicity for dynamic live imaging (Shih et al., 2012). Right now, only cortical live imaging data is available for the neonatal brain but hopefully more brain regions will start to be explored such as visual cortex, deep cortical layers (grey-white matter interface), hippocampus or cerebellum and the new models can be adapted to different regions and their vascular heterogeneity. The multiphoton high-resolution data from adult mice have been coupled with mathematical modelling to investigate flow after vascular diameter increase (Berthiaume et al., 2022; Schmid et al., 2021). However, inferring neonatal cerebrovascular function from adult data should be avoided since the newborn brain is more than ‘a tiny adult brain’ with particular characteristics. We have learned from imaging studies that brain vasculature in infants is extremely dynamic, developing new branches and remodelling existing vessels to fulfil the function of irrigating the brain tissue. Nevertheless, some of the modelling framework used to analyse the adult brain can be translated to the infant brain. For instance, the network segmentation acquired through multiphoton microscopy to obtain the vascular morphology and organisational architecture, and then the implementation of mathematical models of flow and irrigation to follow longitudinally the gain of function in the infant brain. In particular, through the vascular segmentation of the infant brain could be obtain the vascular network architecture and structure (by identifying how the network organises its hierarchy, the connections between vessels and their length, branches points and diameters overtime), point out the inlets and outlets in the network, oxygenation levels, and the vascular distribution within other cells of the brain. Additionally, we can collect information about how other cells such as glial and mural cells modulate the capillary formation and maturation. These data can then be fed into mathematical models to evaluate the vascular network function in infant brains of different ages.

Strikingly, vascular development in tissues has been thoroughly modelled during the last 20 years. Though some angiogenesis mathematical models are focused on cellular traction forces, cell-cell communication and rearrangement during sprout extension (Santos-Oliveira et al., 2015; Gómez-Escudero et al., 2019), several others have simulated the growth of a complete bifurcating vasculature following local gradients of vascular growth factors produced by hypoxic tissue cells (Caforio et al., 2022; Sousa et al., 2021; Xu et al., 2016; Vavourakis et al., 2017). Moreover, models of vascular remodelling and pruning have been developed both at cell level (Loerakker et al., 2018), as well as at vessel and network levels (Pries et al., 2015). Therefore, in tandem with microscopy observation, mathematical modelling of blood flow, angiogenesis and vascular remodelling has the ability to explore the increase in function of the growing vascular network as it develops, leading to a better understanding of the mechanisms of vascular adaptation *in vivo*.

The detailed calculation of blood flow from the network geometry is not trivial as it requires solving the 3D-Navier-Stokes equations inside the volume of the vessels with knowledge of the time dependent pressure profiles at the network inlets and outlets, as well as of the elasticity of the vessel and surrounding tissue (Quarteroni et al., 2016; Oliveira et al., 2020). However, simplifications of these complex calculations are often implemented either to address the propagation of pressure waves along the vessels, through so-called 1-D models (Quarteroni et al., 2016; Caforio et al., 2022), or to calculate the time average blood flow and tissue irrigation (Linninger et al., 2019; Secomb et al., 2013; Shih et al., 2015; Berthiaume et al., 2022). These latter simplified models, also called 0-D models of blood flow, normally consider laminar flow, but importantly may take into account the asymmetric division of hematocrit at the network bifurcations, and the blood viscosity dependence on vessel width (Fåhræus and Lindqvist, 1931; Schmid et al., 2019). In the smallest vessels the blood cannot be considered as a Newtonian fluid as the mechanics of erythrocyte deformation influence flow. Still, 0-D models can take into account both the non-newtonian behaviour of blood and the influence of vessel wall elasticity in flow, if required (Yáñez et al., 2019; Flores et al., 2013). The simplicity of these models has permitted the estimation of blood flow in very large networks as in whole murine brains (Linninger et al., 2019; Hartung et al., 2021). By computing the hematocrit in the network, these models can be coupled with descriptions of oxygen flux along the vessels (Secomb et al., 2013, 2004), so that the transport of oxygen within the tissue can be predicted. In this way, 0-D models have the capacity to identify the regions of low oxygen pressure in the developing cerebral tissue and may be coupled to angiogenesis modelling (Sousa et al., 2021) to predict blood vessel development *in vivo*. Consequently, the observation of vascular development through multiphoton microscopy coupled with irrigation models allows to understand how the growing vascular network operates towards improving the initial deficient irrigation.

At shorter timescales, mathematical modelling can readily analyse the increase in irrigation consequence of localised and rapid alterations in blood vessel diameter (Terman, 2023). For this aim, 0-D models are particularly useful as they predict flow and irrigation in large networks. It is thus possible to use these techniques to compare the irrigation alteration after the increase in diameter of a specific pial arteriole, for example, or of a large number of vessels in the same neighbourhood instead. Vessels’ diameter alterations may be both active (consequence of direct neurovascular coupling and dependent on the contractile mechanics of the mural cells, smooth muscle cells and pericytes, layers in the vessel) or passive (consequence of local alterations in blood pressure). To calculate passive diameter alterations mathematical models have to include the relation between vessel diameter and blood pressure. In the case of arterioles, this requires a detailed implementation of the myogenic effect in the code (Carlson et al., 2008; Flores et al., 2013). In addition, these models can be included into reinforcement learning algorithms that predict the minimal set of vessels needed to increase their diameter to irrigate a specific region after the neuronal activation. By comparing with *in vivo* vascular activity, it will be possible to learn if the adult brain follows an optimal strategy in setting the locations for neurovascular coupling. In fact, we are still learning and uncovering this concept that has divided opinions due some controversy about how the neurovascular coupling evolves and what are the cells and mechanisms involved. And through longitudinal microscopy imaging we may learn how the infant brain gains this capacity for blood flow regulation and what is the influence of glial or mural cells in the neurovascular coupling according to their appearance in development of the neurogliovascular unit.

Cerebrovascular growth and maturation results from a complex network of cellular and acellular interactions across different spatiotemporal scales. Nevertheless, the angio-adaptation models we described above have the limitation of not incorporating the multiscale nature of the cerebrovascular system, which is crucial. There is therefore room for improvement. Coupled with high resolution and longitudinal data collected from multiphoton microscopy, models of vascular remodelling, blood flow and irrigation with reinforcement learning will provide a playground to generate hypotheses and test mechanisms of cerebrovascular expansion and maturation. Following vascular development during the first days after birth will allow to parameterise the irrigation and blood flow models, by identifying the regions of low oxygen delivery where neo-vascularisation and remodelling occurs in a more realistic and biological way. These models can then be used to calculate irrigation after a myriad of combined vessel morphology alterations. Reinforcement learning will identify optimal strategies to increase irrigation locally, as well as the optimal mechanical properties of the vessels that will better adapt to changes in flow.

The challenge in the next few years will be to combine data from cellular interactions during development, since the blood flow as well as the oxygen content are changing at the same time the vascular connections are being made. The combination of these data into models could markedly improve the understanding how vascular dysfunction and malformation leads to neurodevelopmental and neurological diseases. And eventually improve the diagnosis, prognosis as well as the treatment, which is expected to revolutionise personalised medicine in the near future. We are in the right *Era* to build physiological angio-adaptation models using *in vivo* image data from neonatal construction of the cerebrovascular network.
